# Twenty-and-a-half syndrome: a case report

**DOI:** 10.1186/s13256-019-1980-4

**Published:** 2019-02-15

**Authors:** Mukesh Dube, Raunak Dani, Ayush Dubey, Dinesh Chouksey

**Affiliations:** Department of Neurology, Sri Aurobindo Medical College & P.G. Institute, 4th Floor, Sanwer Road, Indore, MP 453555 India

**Keywords:** Twenty-and-a-half syndrome

## Abstract

**Background:**

In the list of named numerical neuro-ophthalmological syndromes, such as one-and-a-half syndrome and others, we report for the first time twenty-and-a-half syndrome, which is characterized by one-and-a-half syndrome with bilateral seventh and right fifth nerve palsy (1.5 + 7 + 7 + 5 = 20.5) in a patient with ischemic stroke.

**Case presentation:**

A 45-year-old Asian Hindu woman presented with vomiting and imbalance of 1 day’s duration. She had left-sided ataxic hemiparesis with one-and-a-half syndrome with bilateral seventh and right fifth nerve palsy. Magnetic resonance imaging of her brain revealed acute non-hemorrhagic infarct in the right posterolateral aspect of pons and medulla, with normal brain vessels angiography. We described her disorder as twenty-and-a-half syndrome. She was put on antiplatelet therapy.

**Conclusions:**

Twenty-and-a-half syndrome is reported for the first time. It is due to posterior circulation stroke; in our case, it was due to lacunar infarcts in the pons and medulla, manifesting as one-and-a-half syndrome with bilateral seventh and right fifth nerve palsy.

## Background

There are many syndromes that describe disorders affecting eye movements and cranial nerves, such as one-and-a-half syndrome, eight-and-a-half syndrome, fifteen-and-a-half syndrome, sixteen-and-a-half syndrome, and twenty-four-and-a-half syndrome. In 1967, C. Miller Fisher reported horizontal gaze palsy to one side along with ipsilateral internuclear ophthalmoplegia (INO) resulting in loss of all horizontal movements in the ipsilateral eye, except abduction of the contralateral eye, with the lesion within the ipsilateral pontine tegmentum, naming it numerically as one-and-a-half syndrome. Anatomically, involvement of a combination of medial longitudinal fasciculus (MLF) plus abducens nerve nucleus or involvement of MLF plus paramedian pontine reticular formation (PPRF) can cause one-and-a-half syndrome. The involvement of a facial nerve with one-and-a-half syndrome was termed eight-and-a-half syndrome by Eggen-Berger who first reported three such cases.

In continuation of this concept, we report a new neuro-ophthalmological syndrome, twenty-and-a-half syndrome, comprising one-and-a-half syndrome with bilateral seventh and right fifth nerve palsy, which has never been described before in the medical literature; furthermore, this novel syndrome gives an insight into the brainstem and its connections, in particular, the dorsolateral pons and medulla.

## Case presentation

**Fig. 1:**
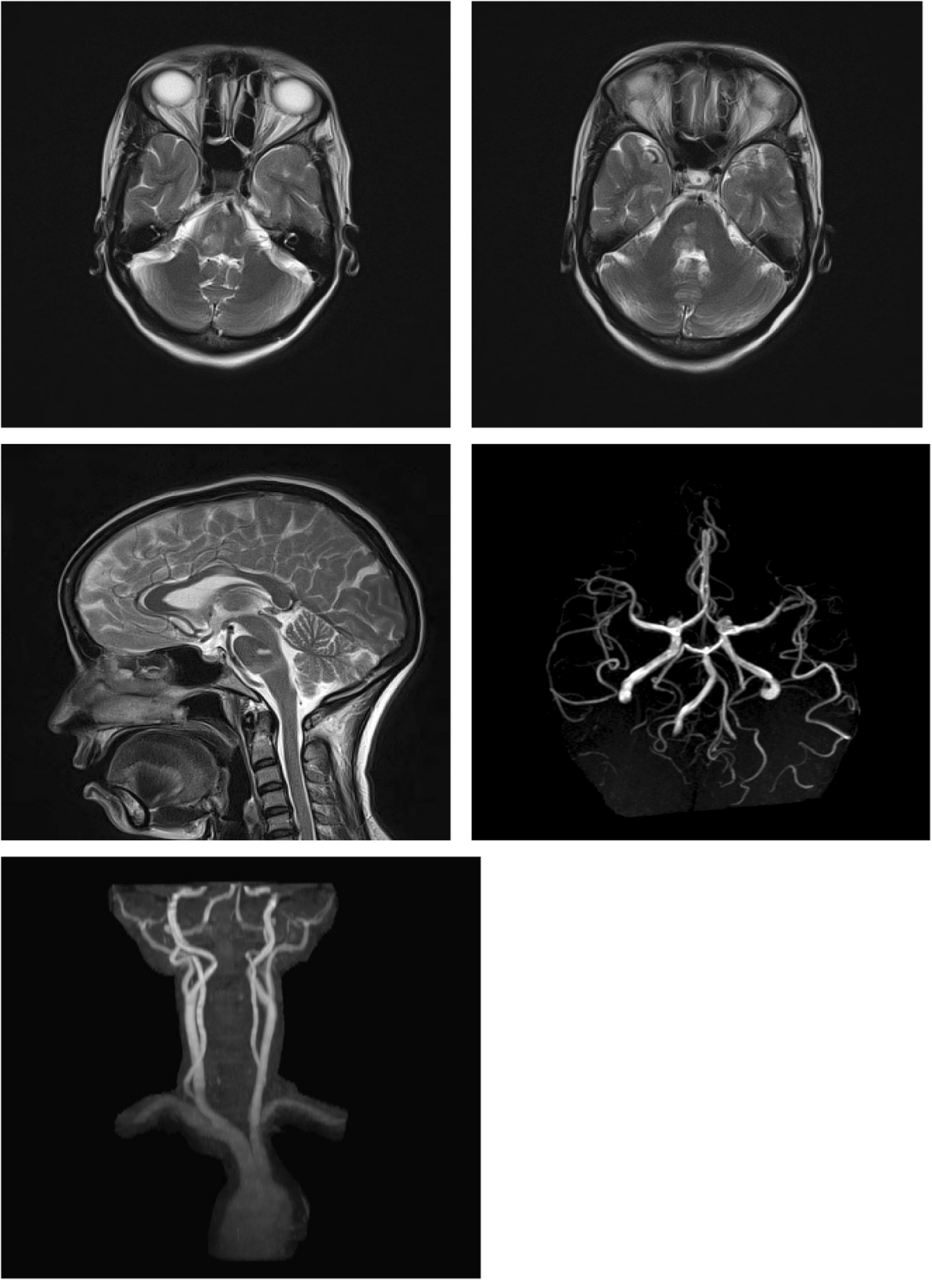
Right paramedian pontine infarct with normal brain angiography

A 45-year-old, right-handed, Asian Hindu woman presented with acute onset dizziness with imbalance and vomiting of 1 day’s duration. She was a known case of hypertension and had a history of complete recovery from a stroke with left hemiparesis 4 years earlier, for which she was put on aspirin 150 mg a day, atorvastatin 10 mg a day, and clonidine 0.1 mg three times a day. She had a non-contributory family history for cardiac and neurological events. She was a housewife, she did not drink alcohol, she did not smoke tobacco, and she belonged to lower economic class; she resided in a rural area, living in a pucca (solid and permanent) house in a clean environment. At the time of admission she was conscious, and oriented to time, place, and person. Her pulse rate was 80/minute, regular, normovolemic, all peripheral pulsations were well felt with no carotid bruits. Her blood pressure was 140/100 mmHg in supine position. She was afebrile. A cranial nerve examination revealed right horizontal gaze and right eye adduction restriction with horizontal nystagmus on abduction of left eye. An absent bilateral corneal reflex and decreased sensation over right half of face along with bilateral lower motor neuron (LMN)-type facial nerve palsy was present. A motor examination revealed left ataxic hemiparesis. An MRI of her brain was done which revealed diffusion restriction and apparent diffusion coefficient (ADC) correlation in the right posterolateral aspect of pons and medulla most likely representing acute non-hemorrhagic infarct with lacunar infarcts suggestive of small vessel ischemia (Fig. 1). A magnetic resonance (MR) angiography of her brain showed normal posterior, anterior circulation, and neck vessels (Fig. 1). A color Doppler of her neck and echocardiography were normal. Her hemoglobin was 10.3, packed cell volume (PCV) 30.9, mean corpuscular volume (MCV) 90.7, total leukocyte count (TLC) 13,500, erythrocyte sedimentation rate (ESR) 11, and serum homocysteine 13.63. The results of antiphospholipid antibodies (APLA) and antinuclear antibodies (ANA) tests were normal. A peripheral smear showed mild hypochromia with anisocytosis. Serum sodium was 136, potassium 3.72, serum creatinine 0.67, blood urea 20, random blood sugar (RBS) 107, serum thyroid-stimulating hormone (TSH) 1.32, total cholesterol 111, high-density lipoprotein (HDL) 31, low-density lipoprotein (LDL) 66, and triglycerides 67.

She was admitted for 10 days, during this time she underwent treatment as well as physiotherapy. She was put on aspirin 150 mg twice a day, atorvastatin 20 mg a day, and ramipril 5 mg once a day. At the time of discharge, she had a modified Rankin Scale (MRS) of 3 with improvement in her ataxic hemiparesis and persistence of the cranial nerve deficits. During her 6-month follow-up she was able to walk without support but still had difficulty in tandem walk. Her facial asymmetry showed marginal improvement. Her corneal reflexes appeared. No further brain imaging was done.

## Discussion and conclusions

We described a patient with right horizontal gaze and right eye adduction restriction with horizontal nystagmus on abduction of left eye suggestive of right INO, absent bilateral corneal reflex, and decreased sensation over right half of her face suggesting right fifth cranial nerve palsy along with bilateral LMN-type facial nerve palsy suggestive of bilateral LMN-type seventh cranial nerve palsy with etiology being ischemic posterior circulation stroke due to small vessel disease (SVD) of the brain.

In this case we propose twenty-and-a-half syndrome characterized by one-and-a-half syndrome with bilateral seventh and right fifth nerve palsy (1.5 + 7 + 7 + 5 = 20.5) due to brainstem infarct in our case. There are similar syndromes described on the basis of the effect on cranial nerves, such as eight-and-a-half syndrome, fifteen-and-a-half syndrome, sixteen-and-a-half syndrome, and twenty-four-and-a-half syndrome. However, to date, twenty-and-a-half syndrome has not been reported and our case adds a new neuro-ophthalmological syndrome to this numerical list.

One-and-a-half syndrome consists of horizontal gaze palsy to one side along with ipsilateral INO, leading to loss of all horizontal movements in the ipsilateral eye with horizontal nystagmus in the abducting contralateral eye, with anatomical localization within the ipsilateral pontine tegmentum wherein there is involvement of MLF with abducens nerve nucleus, or MLF + PPRF. As per C. Miller Fisher, pontine base infarction may produce ataxic hemiparesis as pontocerebellar fibers are widespread at this level and may get easily involved [1]. The clinical findings of our case were explained by involvement of right dorsolateral pons and medulla except left LMN facial palsy. This can be explained by the fact that a diffusion image can be negative in 8% of all patients who have had a stroke, especially in SVD and posterior circulation strokes [2]. The large blood vessels of our patient were normal in MR angiography, and carotid Doppler further supported pure SVD. Lacunar infarcts are small, discrete, often irregular lesions, ranging from 1 to 15 mm in size, frequently located in the putamen and the pallidum, followed by the pons, thalamus, caudate nucleus, internal capsule, and corona radiata. These lesions are not found in the cerebral or cerebellar cortices. On histopathologic examination, these small cavities usually show fine strands of connective tissue resembling cobwebs. The lacunes and microemboli are responsible for infarct in SVD. The underlying vascular brain damage is attributed to obliteration of arterial lumen by the thickened arterial media, obstruction of the penetrating arterial lumen by parent large intracranial artery intimal plaques, or microatheromas arising at the origin of the branch itself. C. Miller Fisher analyzed the lacunes and termed these pathological changes as segmental arterial disorganization and lipohyalinosis [3]. Impaired perfusion in penetrating artery territory secondary to intrinsic occlusive disease of penetrator vessel, along with compromised collateral circulatory capacity of deep penetrator vessel territory or branch artery orifice occlusion by a plaque in parent artery leads to deep small infarcts as well as infarcts larger than the territory of acutely compromised penetrating vessel [3].

This patient behaved in a true manner of small vessel brain disease. Despite being on antiplatelet drugs she had a second stroke. It was thought that antiplatelets have a doubtful role in SVD, but after Cilostazol Stroke Prevention Study (CSPS) [4] that view has been revised. Small vessel ischemic strokes have a recurring nature leading to subsequent strokes not antiplatelet failure [5]. Thus, to conclude, this numerical novelty, twenty-and-a-half syndrome, due to posterior circulation stroke, adds to our existing knowledge in medicine.
